# A Call-to-Action for Clinicians to Implement Evidence-Based Best Practices When Caring for Women with Uterine Fibroids

**DOI:** 10.1007/s43032-022-00877-3

**Published:** 2022-02-17

**Authors:** Nicholas Leyland, Mathew Leonardi, Ally Murji, Sukhbir S. Singh, Ayman Al-Hendy, Linda Bradley

**Affiliations:** 1 Department of Obstetrics and Gynecology, McMaster University, 1280 Main St. West HSC 3 N 21, Hamilton, ON L8S 4K1 Canada; 2 Department of Obstetrics and Gynecology, University of Toronto, Toronto, ON Canada; 3 Department of Obstetrics, Gynecology and Newborn Care, University of Ottawa &, The Ottawa Hospital, Ottawa, ON Canada; 4 Department of Obstetrics and Gynecology, University of Illinois at Chicago, Chicago, IL USA; 5 Department of Obstetrics and Gynecology and the Women’s Health Institute, Cleveland Clinic, Cleveland, OH USA

**Keywords:** Abnormal uterine bleeding, Heavy menstrual bleeding, Hysterectomy, Leiomyoma, Myomectomy, Uterine fibroids

## Abstract

Uterine fibroids are common benign tumors that occur in up to 80% of women. Approximately half of the women affected experience considerable physical, psychological, and economic burdens and impact on quality of life due to symptoms such as heavy menstrual bleeding, pelvic pain, and infertility. Several medical and surgical options are available to treat uterine fibroids; however, healthcare providers may benefit from practical guidance in the development of individualized treatment plans based on a personalized approach. Medical treatments and minimally invasive procedures are generally preferred by most patients before considering more invasive, higher risk surgical interventions. In general, patient-centered, uterine-preserving procedures may be prioritized based on the patient’s goals and the clinical scenario. Occasionally, hysterectomy may be the preferred treatment option for some patients who require definitive treatment. This call-to-action highlights recent challenges to patient care, including radical shifts in physician–patient interactions due to the COVID-19 pandemic and recent changes to evidence-based, clinically approved therapies. This report also reviews contemporary recommendations for women’s health providers in the diagnosis and medical and surgical management of uterine fibroids. This call-to-action aims to empower healthcare providers to optimize the quality of care for women with uterine fibroids utilizing the best available evidence and best practices.

## Introduction

Several key events took place during 2020 and the COVID-19 pandemic that have impacted how physicians diagnose and treat uterine fibroids. The use of virtual medical consultations as well as investigational and procedural delays has increased due to the COVID-19 pandemic [1]. Second, there have been significant changes to clinically available treatment options with the recent US Food and Drug Administration (FDA) approval of the first two oral treatments for uterine fibroids (elagolix with estradiol/norethindrone acetate and relugolix with estradiol/norethindrone acetate) and the withdrawal of the selective progesterone receptor modulator ulipristal acetate in Europe and Canada. Although some evidence-based guidelines [2] have been updated with guidance for the management of uterine fibroids, not all have been updated; thus, more recently available treatment options may not be reflected in existing guidelines. This call-to-action highlights current challenges and limitations to fibroid diagnosis and management and examines newer treatment options to help clinicians make updated, evidence-based decisions to treat patients with uterine fibroids.

## Overview of Disease Burden

Uterine fibroids (also known as leiomyomas or myomas) are benign, monoclonal neoplasms that form in the myometrium and are associated with symptoms such as heavy menstrual bleeding (HMB), bulk-related symptoms (e.g., pelvic pain and pressure), and reproductive dysfunction (e.g., obstetric complications and infertility) [3]. Uterine fibroids are the most common type of benign pelvic tumor in women [4, 5] and are associated with decreased quality of life (QOL) due to negative impacts on social and intimate relationships, physical activity, work productivity, and psychological well-being [5]. Uterine fibroids also lead to significant individual and societal economic impacts, with costs estimated at (US) $9.4 billion per year; these costs are attributed to direct and indirect expenditures associated with obstetric complications, diagnostic- and treatment-related costs, costs of sanitary products, and increased absenteeism and presenteeism [6, 7]. Major risk factors for uterine fibroids include age (up to menopause) and Black race [3, 8]. Women of color are three times more likely to develop fibroids, present at an earlier age, experience more severe symptoms, and have higher rates of hospitalization and surgical intervention (myomectomies and hysterectomies) compared with White women [9, 10]. The disproportionate impact on Black women has wide-ranging implications stemming from known socio-economic disparities and reduced access to healthcare [10]. As a result, uterine fibroid management has a wide-reaching impact on society at large.

## Challenges and Limitations of Uterine Fibroid Management

### Challenges in Diagnosis

Diagnosis of uterine fibroids and selection of treatment options are guided by imaging techniques such as ultrasound, sonohysterography, or magnetic resonance imaging (MRI). However, lack of adherence by physicians to standardized imaging guidelines represents a clinical challenge. In fact, findings from a recent Canadian report show that physicians do not consistently follow the recommendations outlined in the Morphological Uterus Sonographic Assessment consensus statement [11, 12]. In addition, the quality of ultrasound reports falls short of internally endorsed guidelines and requires improvement [12]. Clear, high-quality imaging is required for physicians to determine fibroid size, location, and impact on the endometrial cavity and the endometrium. Standardized quality performance, interpretation, and reporting are essential to inform diagnoses and guide physicians to counsel their patients in choosing appropriate and optimal therapies. Ultrasonography is the most commonly used imaging technique to diagnose and monitor uterine fibroids, owing to the technology’s high availability, ease of use, and low cost [12]; however, inaccurate interpretation of ultrasonography images may lead to misdiagnoses [13]. Sonohysterography is also commonly used to diagnose submucosal fibroids and evaluate the degree of endometrial cavity involvement [14]. Improved imaging guidelines are needed to help physicians more accurately diagnose patients (e.g., distinguish uterine fibroids from adenomyosis or other malignancies), evaluate the uterine cavity, predict therapeutic response, or anticipate potential surgical approaches and challenges during such procedures. Beyond improving the quality of what we currently have access to, newer imaging options include strain elastography and shear wave elastography, which may help guide diagnoses and inform treatment decisions by providing information on tissue stiffness [13, 15].

In addition to improved development and adherence to imaging guidelines and accurate diagnosis, there is a need for wider adoption of these recommendations across the world. The most recent and widely used current classification system, developed in 2011 [16] and modified in 2018 [17] by the International Federation of Gynecology and Obstetrics, is based on fibroid location and degree of intramural/intracavitary extension. This classification system has been widely adopted and is easy to follow; however, the location-based system may not necessarily correlate with tumor biology or patient symptoms [18]. The lack of pathological criteria for non-surgical interventions also presents a diagnostic challenge [19]. Additional research is needed to further characterize and distinguish specific subtypes of uterine fibroids to account for various features of fibroid pathophysiology and more effectively guide individualized diagnoses and interventions.

### Challenges Due to the COVID-19 Pandemic and Virtual Medicine

Physician–patient interactions have changed in recent times due to the shift from in-person consultations to virtual appointments precipitated by the COVID-19 pandemic. Although there are many apparent benefits to telehealth [20, 21], including safety, convenience, and increasing access to care, concerns have been raised around clinical practice patterns for patients in remote settings [22]. In addition, the lack of in-person assessment may limit accurate evaluation or examination of the patient and prevent or delay tissue sampling [1], leading to misdiagnoses or delayed diagnoses, delays in blood testing (e.g., to identify the degree of anemia), or missed identification of acute emergencies such as prolapsed fibroids. Delayed assessment, diagnosis, and intervention may also cause lesions to enlarge and symptoms to progress, negatively impacting patient QOL [23, 24]. In the case of procedural delays due to pandemic-related shifts in hospital policies or patient fears, medical options may help bridge the gap in treatment, reduce the volume of lesions, and optimize hemoglobin levels until such time that the patient is able to undergo surgery. However, surgical guidelines developed during the COVID-19 pandemic recommend that patients with urgent benign gynecological issues and substantial symptoms should not delay surgical intervention [25]. Furthermore, the COVID-19 pandemic has disproportionally affected women of color, who are more likely to contract COVID-19, are at increased risk for hospitalization and mortality due to COVID-19, have reduced access to high-quality healthcare, and are more likely to work outside the home and/or in jobs without paid sick leave compared with White women [26–29].

### Prevention, Diagnosis, and Treatment of Anemia

Another current challenge in treating patients with uterine fibroids is the management of iron deficiency anemia, which is a common comorbidity experienced by approximately two-thirds of women who experience HMB with uterine fibroids [30–33]. The recommended primary prevention strategy for iron deficiency anemia is adequate intake of dietary iron [34]. Anemia can substantially impact QOL and appropriate treatments for iron deficiency are generally associated with improvements in QOL [30, 35, 36]. Oral supplementation with iron salts is commonly used as first-line therapy for the treatment of anemia; if patients do not improve or cannot tolerate iron salts, intravenous iron is recommended [37]. Once anemia is corrected it may require up to 3–6 months to replete iron stores; iron supplementation should continue after anemia resolves. In addition to oral or parenteral iron, medical management that decreases menstrual blood loss is a valuable adjunct method to treat anemia. Appropriate screening and treatment of anemia is recommended to improve symptoms and correct hemoglobin levels before surgery due to the increased risk of postoperative morbidity and mortality associated with anemia [15, 38]. Surgeons should aim for hemoglobin levels > 12.0 g/dL prior to performing elective fibroid surgery [39]. Perioperative blood transfusions can generally be avoided in treating anemia, as transfusions are associated with significant morbidity, including sensitization leading to the potential development of hemolytic disease in the fetus and newborn [38]. The development of clinically significant alloantibodies occurs in approximately 8.1% of patients who receive blood transfusions [38]. Such sensitization is an important issue to prevent in women with fibroids who are planning future fertility. Aggressive treatment of anemia is important, especially before surgery, and can help reduce the need for blood transfusions.

## Management of Uterine Fibroids

### Expectant Management

Many patients with uterine fibroids do not experience any symptoms; in fact, incidental discovery of fibroids is relatively common [31, 33]. For these patients, no treatment may be necessary, and watchful waiting may be considered [31, 40, 41]. However, it is important to tailor interventions for individual patients based on menopausal status, the patient’s desire to preserve fertility, or where there is a suspicion of malignancy. Patient-centered disease management is guided by medical history, physical examination, imaging, and results of blood tests. If patients begin to experience symptoms and desire treatment, a fibroid-specific management plan can be developed as outlined in the following sections.

### Medical Management

**Fig. 1:**
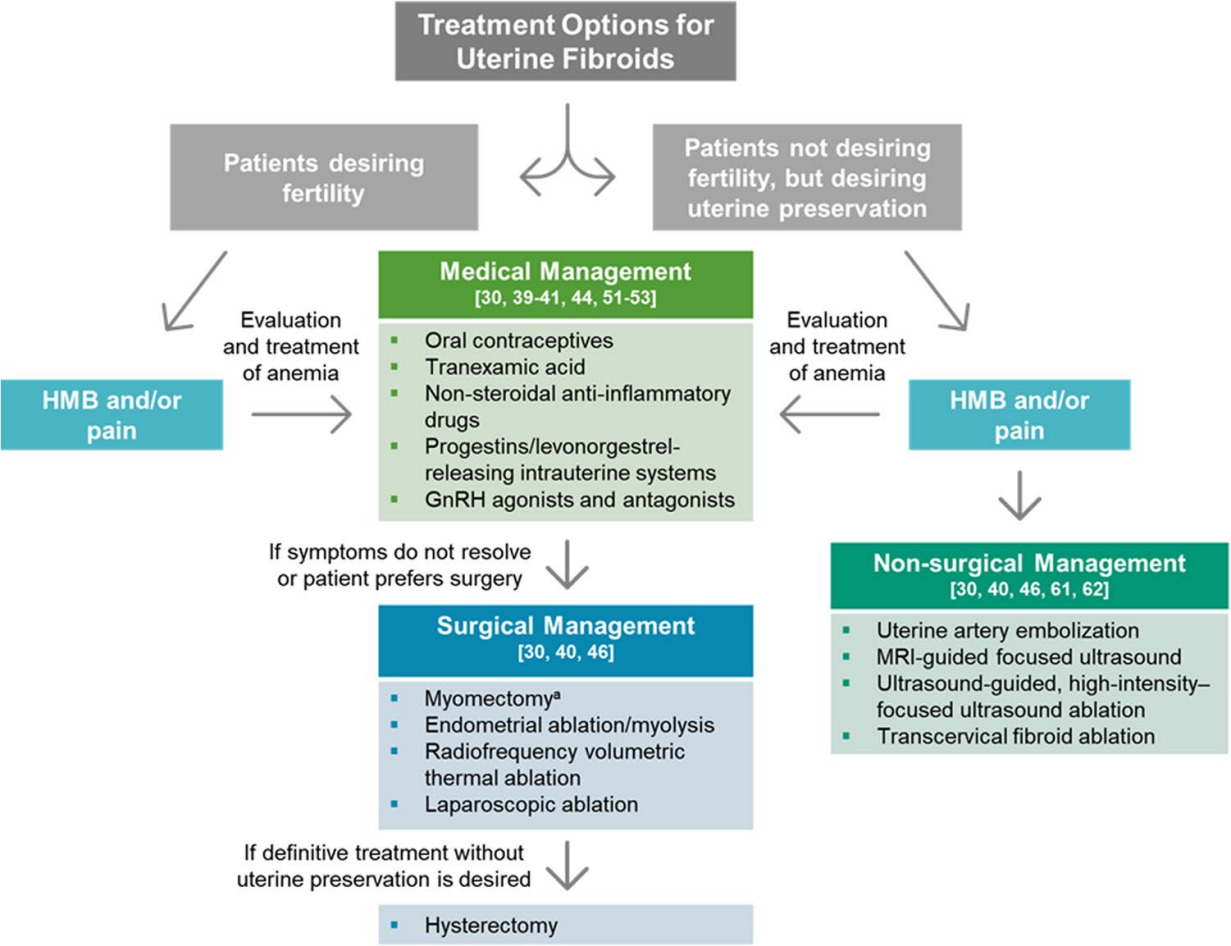
Treatment options for uterine fibroids. Evidence-based treatment decisions should be tailored according to the individual clinical scenario (e.g., size and location of fibroids, patient age, symptoms, desire to preserve fertility, access to therapy) and clinician judgment [41]. ^a^May be performed hysteroscopically, laparoscopically, abdominally, or with robotic assistance. GnRH, gonadotropin-releasing hormone; HMB, heavy menstrual bleeding; MRI, magnetic resonance imaging

Several options for medical management are currently available to decrease uterine fibroid size, reduce menstrual blood loss, improve hemoglobin levels, and/or improve fibroid-associated symptoms (Fig. 1 and Table 1) [31, 40–42]. Not all medical management options provide contraception, but some of them (e.g., oral contraceptives) have that additional benefit. Medical treatments may be used while patients await surgery to manage fibroid size and symptoms or may even replace surgical options in some cases [31]. When given the choice between surgery and pharmaceutical management of uterine fibroids, many women would choose the latter to avoid major surgery or to address other personal preferences [43, 44]. It is important to note that not all the clinical practice guidelines have been updated to include all approved pharmaceutical options for medical management of fibroids. The most recent American College of Obstetricians and Gynecologists practice bulletin on the management of fibroids was published in 2021 [2] and a comparative effectiveness review was published in 2017 by the Agency for Healthcare Research and Quality [7]. The most recent update to the 2015 Society of Obstetricians and Gynecologists of Canada guidelines [33] for medical management of symptomatic fibroids was published in 2019 [45]. Decisions regarding the medical management of uterine fibroids should be guided by the physician’s assessment, appropriately tailored for each patient, and shared between the patient and physician [2, 41, 46]. Evidence-based treatment plans must consider fibroid type (size, location, and characteristics) and patient’s symptoms, medical history, desire for fertility, access to therapies, and impact on QOL [31].

**Table 1:** Medical management of uterine fibroids

Treatment	Evidence-based recommendation
Oral contraceptives (estrogen/progestin)	Reduces HMB but does not inhibit fibroid growth or reduce fibroid volume [31, 40–42]
Tranexamic acid	Non-hormonal oral antifibrinolytic agent; reduces HMB but has no effect on fibroid size; widely available globally [31, 41, 42]
Non-steroidal anti-inflammatory drugs	Reduces HMB and pain, though less effectively than estrogen/progestin contraceptives, the levonorgestrel-releasing intrauterine system, or tranexamic acid [31, 41]
Oral or injectable progestins	Reduces HMB but data supporting effectiveness are limited [2, 41]
Levonorgestrel-releasing intrauterine system	Reduces HMB to a greater extent than oral contraceptives; may have limited benefits in women with high fibroid burden that distorts the uterine cavity due to risk off expulsion [31, 41, 42]
GnRH agonists	Reduces HMB, significantly reduces fibroid size, and improves hemoglobin levels; recommended in combination with low-dose estrogen/progestin add-back therapy to mitigate adverse effects and/or as pretreatment to reduce fibroid volume before surgery (3–6 months) [7, 31, 40–42, 53]
GnRH antagonists	Reduces HMB and fibroid volume; improves hemoglobin levels; recommended in combination with low-dose estrogen/progestin add-back therapy to mitigate adverse effects [31, 42]
Selective progesterone receptor modulators	Reduces HMB, pain, and fibroid volume and increases hemoglobin levels; recommendations suspended in 2020 due to safety concerns; long-term safety is under investigation [31, 42, 56, 57]
Aromatase inhibitors	Limited evidence to demonstrate reductions in HMB or fibroid size [31, 41]
Natural therapy (vitamin D, epigallocatechin gallate)	May inhibit fibroid growth; currently under clinical investigation and further evaluation is needed [31, 58, 59]

*GnRH*, gonadotropin-releasing hormone; *HMB*, heavy menstrual bleeding

Currently available hormonal therapies for the treatment of uterine fibroids include combined estrogen/progestin oral contraceptives, progestin-only contraceptives (including the progestin-(levonorgestrel-) releasing intrauterine system [LNG-IUS]), and gonadotropin-releasing hormone (GnRH) agonists and antagonists. Combined estrogen/progestin oral contraceptives are commonly used as first-line therapy for the treatment of uterine fibroids; these agents reduce fibroid-associated symptoms such as HMB, but do not inhibit fibroid growth or reduce fibroid volume [31, 40–42]. Oral or injectable progestins can reduce HMB, but they are not currently recommended to treat uterine fibroids because there is a lack of sufficient evidence to prove their effectiveness [40, 41]. Other non-hormonal options, such as the antifibrinolytic agent tranexamic acid or non-steroidal anti-inflammatory drugs, also reduce HMB and other fibroid-associated symptoms, but do not inhibit fibroid growth or reduce fibroid size [31, 40–42]. Non-steroidal anti-inflammatory drugs are less effective in reducing HMB than tranexamic acid, combined estrogen/progestin oral contraceptives, or the LNG-IUS [31, 41].

The LNG-IUS acts locally on the endometrium to decrease HMB (leading to amenorrhea or improvements in menorrhagia) and reduces HMB to a greater extent than do oral contraceptives [31, 41, 42, 47]. A systematic review of 10 studies evaluating the LNG-IUS to treat uterine fibroids demonstrated substantial reductions in HMB; some studies showed reductions in uterine and fibroid volumes, while others showed no change [47, 48]. The LNG-IUS is indicated for long-term, reversible contraception and HMB, but is contraindicated for women with a high fibroid burden that distorts the uterine cavity. Risk of LNG-IUS expulsion is higher in women with fibroids; reported expulsion rates are between 0 and 20% [31, 41, 42].

GnRH antagonists are the most recent addition to the options for the treatment of uterine fibroids [40]. Elagolix, an oral GnRH receptor antagonist, administered in combination with hormonal add-back therapy (estradiol 1 mg and norethindrone acetate 0.5 mg once daily; E2/NETA) is the first FDA-approved (May 2020) oral treatment option for uterine fibroids and is indicated for the management of HMB associated with fibroids in premenopausal women for up to 24 months. The Elaris Uterine Fibroids 1 and 2 studies were identically designed as 6-month, phase 3 randomized trials that evaluated the efficacy and safety of elagolix with add-back therapy in women with fibroid-associated HMB. Findings from these studies indicated that patients taking elagolix with add-back therapy experienced significantly less HMB associated with uterine fibroids compared with patients taking placebo [49]. Elagolix is also highly effective in decreasing fibroid volume [31, 50]; however, the effects of elagolix on fibroid size are diminished with add-back therapy. Another GnRH antagonist, relugolix, was approved by the FDA in May 2021 in combination with E2/NETA for the management of HMB in premenopausal women. This approval followed the recent publication of two 24-week randomized, placebo-controlled, phase 3 clinical studies that demonstrated significantly higher reductions in fibroid-associated HMB and pain, reduced uterine volume (suggesting decreased fibroid burden), and increased hemoglobin levels with relugolix and E2/NETA compared with placebo [51]. Findings from a recent phase 2b study indicated that linzagolix significantly reduced endometriosis-associated pain [52], and linzagolix is currently being evaluated in clinical trials to treat HMB associated with uterine fibroids and reduce fibroid volume [42]. This class of drugs has the potential to significantly improve the management of acute and chronic HMB; however, they may be less available and/or more expensive in countries without universal access to healthcare.

Results from recent studies have also shown that the GnRH agonists leuprolide, goserelin, and triptorelin decrease fibroid volume, reduce HMB and fibroid-associated pain, and increase hemoglobin levels [31]. A Cochrane review [53] examined findings from multiple randomized controlled trials and found clear evidence that GnRH analogs (both antagonist and agonists) can improve hemoglobin levels and reduce fibroid and uterine volume. One challenge with these agents is the appropriate administration with respect to the patient’s menstrual cycle to avoid a hormonal “flare” effect and associated increased HMB.

GnRH agonists and antagonists have been associated with adverse effects such as hot flushes, decreased libido, and sleep disturbances; however, many of these effects can be successfully alleviated with hormonal add-back therapy [31, 42]. Loss in bone mineral density (BMD) has also been associated with GnRH agonists and antagonists; evidence from the Elaris [49] and Liberty [51] studies demonstrated that even with add-back, BMD loss is observed and long-term implications of this are currently unknown. Addition of add-back therapy does slightly reduce the beneficial effects of GnRH analogs on menstrual blood loss and fibroid volume; however, the benefits of adding add-back therapy for the management of uterine fibroids are important to consider.

Selective progesterone receptor modulators were initially considered promising treatment options and effectively reduced fibroid volume, treated symptoms, and delayed or eliminated surgical intervention [54]. However, these therapies have recently been scrutinized because of safety concerns, specifically the rare occurrence of liver dysfunction leading to liver failure [40, 55]. In several clinical trials, ulipristal acetate has demonstrated significant reductions in fibroid volume and HMB along with improvements in hemoglobin levels and was approved in Canada and Europe for medical management of uterine fibroids [31, 42]. However, ulipristal acetate was withdrawn in Canada and Europe in 2020 after cases of drug-induced liver toxicity were reported [42, 56, 57]. In the USA, ulipristal acetate is no longer under clinical evaluation nor is FDA approval being sought for its use in treating symptomatic uterine fibroids. The overall risk of liver injury leading to transplantation was extremely low (six of over 1,000,000 cases), but these cases were not predictable or related to dose [42]. Other selective progesterone receptor modulators including mifepristone, asoprisnil, vilaprisan, and telapristone acetate have also demonstrated efficacy in reducing fibroid-associated symptoms in randomized controlled trials, but clinical investigation is currently paused [31, 56]. Other agents under investigation for the medical management of uterine fibroids include cabergoline, gestrinone, somatostatin analogs, and natural compounds such as vitamin D and green tea extract (epigallocatechin gallate) [31, 58, 59].

### Surgical Management

For more than 100 years, surgical interventions (myomectomy or hysterectomy) have been the main options available to treat uterine fibroids (Table 2); however, surgical procedures may be associated with a high psychological and economic burden, and there is a risk of recurrence of uterine fibroids with uterine-sparing procedures [46]. Hysterectomy is the most common surgical management option and the only method to definitively stop fibroid-associated HMB [30, 31, 41]. Although hysterectomy is associated with risk of perioperative morbidity, in certain cases, it may be the best treatment option in patients who have completed childbearing, and/or have had multiple recurrences of uterine fibroids following other medical or surgical interventions and desire definitive treatment. If surgical management is recommended, referral to high-volume surgeons and medical centers is advised to improve outcomes, decrease length of surgery, and decrease risk of complications. Patients undergoing surgery also benefit when enhanced recovery protocols are employed to improve recovery and patient experiences.

**Table 2:** Surgical management of uterine fibroids

Treatment	Evidence-based recommendation
Hysteroscopic myomectomy	Decreases and removes intracavitary fibroids and improves symptoms; typically preserves the integrity of the myometrium; recommended for FIGO 0, FIGO 1, and some FIGO 2 submucosal fibroids and for patients desiring to retain fertility; associated with a 15–50% risk of recurrence [31, 41, 46]
Abdominal myomectomy (laparoscopic, robotic, or laparotomic)	Reduces uterine volume and improves symptoms; recommended for intramural, subserosal, and very large submucosal fibroids that are not amenable to hysteroscopic resection [31]
Endometrial ablation/myolysis	Reduces HMB; uses electrical energy, cryotherapy, heated saline, or radiofrequency energy to destroy the endometrium; recommended for premenopausal patients who do not desire future fertility [31, 41, 46]
Radiofrequency volumetric thermal ablation	Minimally invasive; reduces fibroid volume and improves symptoms; impact on fertility requires further investigation [31]
Hysterectomy	Advised for patients who desire definitive treatment for symptomatic fibroids; should be performed minimally invasively when possible [31, 41, 46]

*FIGO*, International Federation of Gynecology and Obstetrics; *HMB*, heavy menstrual bleeding

Whenever possible, patients should be made aware of less invasive and uterine-sparing procedures, which may minimize the risk to the patient. These include myomectomy via minimally invasive techniques when appropriate for fibroid removal and improvement of bulk symptoms or other less invasive techniques such as endometrial ablation or radiofrequency volumetric thermal ablation to control bleeding [31, 60]. Myomectomy is recommended for patients wishing to preserve fertility; hysteroscopic myomectomy is generally recommended for submucosal fibroids, while laparoscopic or laparotomic myomectomy is advised for intramural and subserosal fibroids [31].

### Non-surgical Management

Other less invasive non-surgical alternatives include uterine artery embolization to interrupt blood flow to the uterus and fibroids and MRI-guided, high-intensity-focused ultrasound to induce fibroid necrosis and regression (Table 3) [31]. Ultrasound-guided, high-intensity-focused ultrasound ablation has also been shown to improve fibroid symptoms and reduce fibroid volume without permanent adverse effects [61]. These techniques are often effective for symptom control and are associated with shorter recovery times and fewer complications compared with surgery, but may not be good options for patients desiring to retain fertility [31]. Another novel minimally invasive approach not yet included in guidelines for the treatment of uterine fibroids is transcervical fibroid ablation, a uterus-preserving technique that locates fibroids using intrauterine ultrasound and treats them with radiofrequency energy [62]; this outpatient procedure has been shown to be safe and effective in multiple studies [62] but is not yet widely available. The challenge with all the aforementioned procedures is the lack of pathological diagnosis with such interventions.

**Table 3:** Non-surgical management of uterine fibroids

Treatment	Evidence-based recommendation
Uterine artery embolization	Minimally invasive; reduces symptoms and decreases fibroid volume by limiting blood supply to the fibroids as non-involved uterus is spared; recommended for patients who are not good surgical candidates or who choose to avoid surgery; may impact uterine and ovarian function; impact on fertility requires further investigation [31, 41, 46]
MRI-guided-focused ultrasound	Minimally invasive yet effective for controlling symptoms and reducing fibroid size; recommended for patients who are not good surgical candidates or who choose to avoid surgery; impact on fertility requires further investigation [31, 41, 46]
Ultrasound-guided, high-intensity-focused ultrasound ablation	Reduces fibroid symptoms and decreases fibroid and uterine volume with no reported permanent adverse effects [61]
Transcervical radiofrequency ablation	Minimally invasive; uses radiofrequency energy to ablate fibroids; not yet included in treatment guidelines [62]

*MRI*, magnetic resonance imaging

## Conclusions

When considering treatment plans for patients with uterine fibroids, important first steps include recognition, acknowledgment, and validation of symptoms; appropriate investigations (including imaging) to diagnose and classify specific fibroid subtypes; and consideration of individual patient needs (including effects on QOL or access to specific interventions) to guide management. This call-to-action highlights challenges and limitations associated with uterine fibroid management; discusses newer options, based on the best available evidence, currently available for the medical management of uterine fibroids; and serves as a guide for physicians to select the appropriate treatment based on individual patient characteristics and needs. Medical options and minimally invasive treatments are generally recommended as first steps for the treatment of uterine fibroids, with other surgical options available if symptoms persist or if the patient prefers surgery as first-line treatment.

## Data Availability

Not applicable.
